# Australian Children's Exposure to, and Engagement With, Web-Based Marketing of Food and Drink Brands: Cross-sectional Observational Study

**DOI:** 10.2196/28144

**Published:** 2021-07-12

**Authors:** Bridget Kelly, Rebecca Bosward, Becky Freeman

**Affiliations:** 1 Early Start, School of Health & Society Faculty of the Arts, Social Sciences and Humanities University of Wollongong Wollongong Australia; 2 Prevention Research Collaboration School of Public Heath, Faculty of Medicine and Health University of Sydney Sydney Australia

**Keywords:** food, beverage, marketing, online, digital

## Abstract

**Background:**

Food is one of the most frequently promoted commodities, and promoted foods are overwhelmingly unhealthy. Marketing normalizes unhealthy foods, creates a positive brand image, and encourages overconsumption. Limited research is available to describe the extent of food marketing to children on web-based media, and measuring actual exposure is challenging.

**Objective:**

This study aims to monitor the extent of children’s exposure to web-based media food marketing as an essential step in increasing the accountability of industry and governments to protect children.

**Methods:**

Children aged 13-17 years were recruited from October 2018 to March 2019. Children recorded their mobile device screen for 2 weekdays and 1 weekend day any time they visited relevant web-based platforms. After each day, the participants uploaded the video files to a secure server. Promoted products were defined using the World Health Organization European Region nutrient profile model.

**Results:**

The sample of 95 children uploaded 267.8 hours of video data. Children saw a median of 17.4 food promotions each hour on the internet. Considering the usual time spent on the internet on mobile devices, children would be exposed to a median of 168.4 food promotions on the web on mobile devices per week, 99.5 of which would not be permitted to be marketed based on nutrient profiling criteria. Most promotions (2613/4446, 58.77%) were peer endorsed and derived from third-party sources.

**Conclusions:**

Exposure to brand content that is seemingly endorsed by peers or web-based communities likely heightens the effects of marketing on children. Regulations to protect children from this marketing must extend beyond paid advertising to paid content in posts generated through web-based communities and influencers.

## Introduction

### Background

Protecting children from the impacts of unhealthy food and beverage marketing has been repeatedly identified at the highest levels of global policy agenda setting as a priority intervention for childhood obesity prevention. The report from the World Health Organization (WHO) from the Commission on Ending Childhood Obesity explicitly urged governments to regulate the marketing of unhealthy food to protect children from obesity and related noncommunicable diseases [1]. The commission called for the implementation of previous WHO recommendations to restrict the exposure and power (persuasive content) of food marketing across all platforms and settings where children gather [2]. Robust empirical and review evidence indicates that children’s exposure to food marketing leads to a cascade of effects, including food brand awareness, positive brand attitudes, and purchase and consumption behaviors [3]. As promoted foods and beverages are almost exclusively for products high in added fat, sugar, and salt (*unhealthy* foods) [4], this may contribute to excess energy intake and poor dietary patterns [5].

For more than a decade, evidence on children’s exposures to, and the impacts of, food marketing has identified the increasing prominence of digital or web-based media marketing [6].

Almost all Australian adolescents aged 13-18 years (94%) have their own mobile screen devices, and three-fourths have a social media account [7]. Most primary school–aged children also have their own mobile devices (67%), whereas 1 in 6 has a social media account. Adolescents spend an average of 43.6 hours at home in front of screens each week, most frequently engaged in web-based social media [7]. This includes screen time for entertainment, communication, and education. Screen time is lower for younger children, with an average of 31.5 hours per week [7] but still greatly in excess of screen time recommendations. Australian guidelines advise limiting recreational screen time to no more than 2 hours per day for children and adolescents [8].

With the advancement of the internet as a social and participatory space, marketers have been able to target and engage users with personal communications, infiltrate web-based communities with brand content, and enable peer endorsement of brand messages [9]. This means that brands can be embedded in web-based content and distort boundaries between marketing and editorial, entertainment, and personal material, diminishing children’s capacity to recognize marketing as a paid promotion [10-12]. Crucially, web-based marketing is tailored to the unique characteristics and preferences of users, using data analytics that include users’ personal information, browsing history, geolocations, and social media engagement [13]. This *behavioral targeting* of marketing on the internet has major implications for various aspects, including children’s privacy, the impact of marketing on children, web-based marketing regulation, and monitoring of children’s web-based marketing exposure to describe and understand the problem [13-15]. The immersive and interactive nature of web-based media and related marketing likely means that the effects of web-based food marketing on children are greater than those of other offline marketing [9,16].

Research evidence on the impact of web-based food marketing on young people has predominately focused on digital games, specifically *advergames* or branded games [17]. A meta-analysis of experimental studies examining the effect of exposure to web-based branded games on children’s attitudes, choices, or intake of unhealthy foods identified a significant positive effect of small to moderate size [18]. Less evidence is available on the effects of other forms of web-based food marketing. In an earlier cross-sectional survey by the authors, we identified that children who reported higher web-based engagement with food brands and content, particularly through web-based videos, were more likely to consume unhealthy foods and drinks [19]. A systematic review of the evidence on the effects of web-based marketing of risk-associated products, including unhealthy foods, alcohol, and tobacco, indicated an association with children and young adults’ attitudes toward, and intended and current use of, these products [20]. In interactive social contexts, including social media, child-brand interactions may be more influential in influencing children to form preferences for, and perceptions of, brands [21].

Monitoring children’s exposure to food marketing is necessary for engaging policy makers and civil society on the issue, holding industries accountable for their marketing practices, and measuring the effectiveness of any regulations and compliance [22]. The International Network for Food and Obesity/noncommunicable diseases Research, Monitoring and Action Support (INFORMAS) is an international consortium of public interest organizations and researchers that seeks to support the generation of monitoring intelligence to describe food environments, including food marketing, and related policy responses. Global monitoring of children’s exposure to food marketing across media platforms and settings, including television, has been undertaken [23]. However, the individualized nature of behavioral targeting of web-based marketing complicates efforts to monitor children’s exposure to food marketing on web-based media.

### Objective

This study aims to quantify and describe children’s exposure to food and beverage marketing during their time spent on the internet, including the types of foods and beverages promoted and the platforms from where exposures were derived. We also sought to describe the nature of promotions, including the extent to which these were found in paid advertising space, on food companies’ own sites and pages, or transmitted through web-based social networks. The approach used to capture marketing exposures also allowed us to identify the extent of children’s engagement or interaction with food promotions. We hypothesized that children would be exposed to a high volume of unhealthy food and beverage marketing in their usual web-based interactions, which exceeded the number of promotions that they see for healthy choices, and that a large proportion of marketing would be peer endorsed and skewed toward third-party sources, such as shared content and blogs. Children aged 13-17 years were selected for the study, as they were deemed to have sufficient cognitive capacities to undertake the web-based survey and monitoring aspects of this project and to comprehend the ethical and privacy considerations of participating. Adolescents are also key social media users and targets for web-based food marketing [13]. Adolescents are susceptible to unhealthy food marketing despite their increasing cognitive ability, and they may be more impulsive in their purchase decisions [24].

## Methods

### Sampling and Recruitment

The study was approved by the University of Wollongong Human Research Ethics Committee (HREC 2018/158). Children were recruited through the national adolescent survey panel of the market research agency McNair yellowSquares. This panel comprises parents of young people across Australia who have agreed to be contacted to participate in research studies (approximately 15,000 panel members). Panel members with children aged 13-17 years were invited to indicate their interest in participating in this study. Interested parents and children were sent the participant information sheets and consent forms to both sign and return. Participants were then asked to complete a short prestudy questionnaire that assessed their eligibility to participate, along with collecting information on their usual time spent on the internet on a mobile device and also on desktop and laptop computers, split by weekdays and weekend days. To be included in the study, children needed to have at least one social media account, log on to social media at least once per day, and have access to a mobile device (phone or tablet) that was compatible with the screen recording apps or settings. Only one child per family was chosen for participation. Participants were recruited in 2 rounds to avoid the school holiday period—October to November 2018 and February to March 2019. A sample size of approximately 150 children was sought from a national population estimate of approximately 1.4 million adolescents [25]. This was based on a margin of error of 5% for estimates of the average number of exposures to unhealthy food or beverage web-based promotions per day with a conservative population variance of 1000.

### Procedure

#### Piloting

The study required children to record and upload data on their internet use on mobile devices and complete pre- and poststudy questionnaires. The main study was preceded by a pilot study of 26 children. The pilot led to major changes in the recruitment strategy (eg, increasing compensation for participant time), participant tracking and reminders, data coding, and improvements to the data upload server.

#### Screen Recording

Each participant was asked to video record their mobile device screen for 2 weekdays and 1 weekend day anytime they went onto relevant web-based platforms or apps. Relevant platforms include social media websites or apps, video sharing websites or apps, or browsing on the internet. They were asked not to record their screen when they were using any banking platform, using personal messaging (eg, SMS, Facebook Messenger, WhatsApp, or personal messaging on Snapchat or Instagram), making phone calls, or browsing through photos in their device’s gallery. Participants nominated which days they would record within a 2-week period of entry into the study. They were sent 3 SMS text messages on nominated days as a reminder to record their screens.

Participants were provided with detailed written instructions and an instructional video to complete the study screen recording and upload tasks. The recording process varied across mobile device operating systems. For Android devices, participants were asked to download an app called the *Lollipop screen recorder*. The iOS participants had to move the screen recording setting of the control panel of their device. Participants could turn the recording on and off through this app or setting. The device showed a symbol at the top of the screen to indicate that the screen was being recorded. This recording function captures all user actions on their mobile device, such as scrolling, typing, and clicking.

#### Data Upload

Each participant was sent a unique log-in link to the McNair yellowSquares web-based database to upload the data. This was a bespoke platform for uploading files, completing questionnaires, tracking participants’ study progress, and communicating any data issues. After each day of recording, the participants were instructed to upload their video files to the database. Participants were encouraged to edit videos using the video editing function on their device and to remove any footage they did not want the researchers to view. Given the size of the video files and the number of uploads being attempted simultaneously, upload to the database experienced issues with slow uploads and file corruption (inoperable files). Consequently, midway through data collection, new participants were instructed to submit their videos using WhatsApp. WhatsApp uses end-to-end encryption and does not store messages on its own servers.

The participants’ video uploads were monitored daily during the data collection period. Data were deemed to be acceptable if the total duration of uploaded videos for the day was at least 30% of the reported usual time on the web on mobile devices (for weekdays and weekend days separately; from the prestudy questionnaire). When participants failed to reach this threshold of recording, they were contacted by email and phone, given further instruction, and asked to complete a replacement day. Participants received Aus $50 (US $38), paid into their research panel account, if they completed all 3 days of data recording. They received Aus $20 (US $15) if they only completed 1 or 2 days of recording. Participants were included in the final sample if they had at least one acceptable weekday and one weekend day.

Although there was minimal risk involved in participation, some of the main ethical concerns in the project were related to potential risks to privacy and confidentiality. Measures were taken to protect the privacy of the participants and to ensure data security.

#### Coding of Video Data

At the end of the study, all video data were transferred to CloudStor, a secure cloud storage server. Each video was watched at least twice by 1 person from a pool of 3 trained research assistants. In the first viewing of the video, all food and beverage promotions (including food and beverage products, retailers, and services) were identified and coded. The second viewing focused on recording the length of time spent on different platforms. Only branded food promotions were captured, including branded products and packages, brand logos, and brand characters. To be included, promotions needed to be shown onscreen for a minimum of 1 second and at least half of the brand name or logo needed to be visible.

The coding frame captured both the frequency and duration (seconds) of promotions onscreen, the nature of these promotions, and any participant engagement. Promotions were classified according to the platform (app or website) on which they occurred and the extent to which participants engaged with the promotion by *liking*, *sharing*, *commenting*, or *clicking* on a link. Promotions were also classified as *paid*, *owned*, or *earned* media [26,27]. Paid media includes promotions generated by the food company, which pays to place these on third-party platforms. Examples include banner advertisements, paid search advertisements, and sponsored posts on social media. Owned media includes food brands’ own websites, blogs, and social media pages. Earned media refers to promotions that do not directly come from the brand but are shared by third parties through reviews, reposts, blogs, referrals, and word-of-mouth.

Promoted products were defined using the WHO Regional Office for Europe nutrient profile model [28]. The WHO model designates products as *not permitted* or *permitted* to be advertised to children based on the thresholds for negative nutrients and energy content. Marketing for food companies, retailers, and restaurants that do not promote specific food products are not covered by the model. As there were a large number of company brand–only promotions for food retailers and restaurants that could not be classified using the WHO model, we also used the INFORMAS food classification system for monitoring food promotions (Table 1). This system classifies food into 3 broad categories—core or healthy, noncore or unhealthy, and miscellaneous—and 37 smaller food groups. Food and beverage retailers, restaurants, and delivery services were variously classified as noncore or miscellaneous, depending on whether they promoted a specific product and the nature of that product.

Interrater reliability was assessed with each research assistant independently coding the video data of the same 6 participants (15 days). The intraclass correlation coefficient was calculated for absolute agreement between the raters, giving an intraclass correlation coefficient of 0.97, indicating excellent reliability. Reliability results were discussed among the research team, and all issues were resolved before continuing. Reliability testing helped to refine the coding rules about the threshold of time, and the visibility of the brand, onscreen for the promotion to be counted.

**Table 1:** Frequency of food and beverage promotions in sample recordings (N=4446).

Food category		Frequency of promotions, n (%)
**Core or healthy foods**		108 (2.43)
	Plain breads, rice, noodles, and crackers	20 (0.45)
	Fruits and fruit products without added fats, sugars, or salt; ≥98% fruit juices	19 (0.43)
	Milks and yogurts (≤3 g fat/100 g), cheese (≤15 g fat/100 g), and alternatives	18 (0.4)
	Bottled water	15 (0.34)
	Low sugar or high fiber breakfast cereals (<20 g sugar and >5 g dietary fiber/100 g)	15 (0.34)
	Meat and alternatives, including unsalted nuts, seeds, and their pastes	10 (0.22)
	Vegetables and vegetable products without added fats, sugars, or salt	4 (0.08)
	Low fat or salt meals: frozen or packaged meals (≤6 g saturated fat and <900 mg sodium per serve), soups (<2 g fat/100 g, exclude dehydrated), sandwiches, and mixed salads	3 (0.07)
	Healthy snacks: based on core foods (<600 kJ and <3 g saturated fat and <200 mg sodium per serve)	3 (0.07)
	Oils high in mono- or polyunsaturated fats	1 (0.02)
**Noncore or unhealthy foods**		2579 (58.01)
	Chocolate and confectionery	539 (12.12)
	Fast food restaurant or delivery service: unhealthy options	503 (11.31)
	Sugar-sweetened beverages	435 (9.78)
	Alcohol	244 (5.48)
	Sweet breads, cakes and biscuits, and high-fat savory biscuits and pastries	165 (3.71)
	Savory snack foods with added salt or fat include chips, extruded snacks, flavored popcorn, and salted or coated nuts	155 (3.48)
	Local restaurant or delivery service: unhealthy options	142 (3.19)
	Supermarket or retailer: unhealthy options	85 (1.91)
	Ice cream and iced confection	84 (1.88)
	Other high-fat or salt products include spreads with added salt, animal fats, high-fat savory sauces (>10 g fat/100 g), and soups (>2 g fat/100 g, dehydrated)	75 (1.68)
	High-sugar or low-fiber breakfast cereals (>20 g sugars or <5 g dietary fiber/100 g)	34 (0.76)
	Full cream milk and yogurts (>3 g fat/100 g) and cheese (>15 g fat/100 g, high-salt cheeses) and alternatives	32 (0.72)
	Flavored or fried instant rice and noodles	37 (0.83)
	Sweet snack foods include sugar-coated dried fruits or nuts and nut- or seed-based bars	14 (0.31)
	Fruit juice or drinks with <98% fruit	13 (0.31)
	Meat and alternatives processed or preserved in salt	12 (0.27)
	High-fat or salt meals: frozen or packaged meals (>6 g saturated fat or >900 mg sodium per serve)	10 (0.22)
**Miscellaneous**		1759 (39.56)
	Fast food restaurant or delivery service: no specific product	931 (20.94)
	Local restaurant or delivery service: no specific product	365 (8.21)
	Supermarket or retailer: no specific product	207 (4.66)
	Local restaurant or delivery service: only healthier options	111 (2.49)
	Tea and coffee	51 (1.15)
	Dietary supplements and sugar-free gum	26 (0.58)
	Fast food restaurant or delivery service: only healthier options	25 (0.56)
	Supermarket or retailer: only healthier options	22 (0.49)
	Recipe additions: include soup cubes, seasonings, and other sauces	19 (0.43)
	Food manufacturer: no specific product	2 (0.04)

#### Pre-Post Questionnaires

Participants were sent a unique link to a web-based questionnaire at the start and end of the study. This captured data on their usual time spent on the web on mobile devices and on all devices on weekdays and weekend days, social media use (on which platforms they had accounts, number of people per pages they followed on each account, and number of food brands they followed), number of food or beverage brand apps they had on their device, and number of emails or SMS messages they received each week from food or beverage companies.

### Analyses

Statistical analyses were conducted using SPSS for Windows, version 25 (IBM Corporation). Data were analyzed descriptively, including the types of promotions (*owned*, *earned*, or *paid*) and nature of promoted foods. The rates of promotions (promotions per hour) were calculated based on the number of promotions on a sampled day divided by the total relevant video duration for that day. Relevant time spent on the internet included that which captured web-based use, excluding personal messaging and banking. The average hourly rate over the 3 days for each person was then calculated and weighted by day type (weekdays and weekend days). The reported usual time spent on the internet was used to extrapolate the rates of marketing during the recorded period to weekly exposures. On the basis of visual inspection of the data and the Shapiro-Wilk test of normality, the rates of web-based food marketing did not meet normality assumptions, and therefore, medians and IQRs were reported. The Kruskal-Wallis one-way analysis of variance with post hoc test and Bonferroni correction was used to compare the median rates of promotions across web-based platforms. Negative binomial regression was used to identify factors associated with higher weekly exposure to promotions. Independent variables included participant age, usual weekly time spent on mobile devices, number of accounts followed on social media, number of food brands followed on social media, and number of food apps on mobile devices. Negative binomial regression was used because the distribution of the outcome had greater variability than expected under a Poisson distribution. The sample mean of the dependent variable (210.0) was substantially smaller than its variance (43,813.1), and the dispersion parameter was 0.593 with a 95% CI that did not include zero, indicating overdispersion.

## Results

### Sample Description

The final sample of 95 children uploaded 272.8 hours of recordings, of which 267.8 hours were relevant (captured web-based use, excluding personal messaging and banking). The study completion rate was 14.8% (95/644). Across the 2 rounds of recruitment, 736 people were disqualified based on the prescreening questionnaire. Furthermore, 429 people declined to participate or did not start the task after qualifying, 95 dropped out during the study, and 25 were excluded as they did not reach the 30% video upload threshold of reported usual time on the web on mobile devices. Across the 280 days of recordings captured, 23% (22/95) reached a threshold of 75%-100% of the usual recorded time spent on the internet, 45% (43/95) captured 50%-74% of the usual time spent on the internet, and 32% (30/95) captured less than 50% of the usual time spent on the internet. Table 2 shows the characteristics of the study participants. Approximately half of the sample lived in suburbs classified as having a high socioeconomic status, based on the Australian Bureau of Statistics Socioeconomic Indices for Areas. Children reported usually spending an average of 12 hours on the web each week on mobile devices and almost 30 hours on the web each week across all devices. Almost all children held accounts on Instagram; in addition, most also had accounts on Facebook, Snapchat, and music streaming apps.

**Table 2:** Sample description (n=95).

Child characteristics			Statistics
Age (years), mean (SD)			16.2 (1.07)
Usual weekly web-based media use mobile devices (hours), mean (SD)			12.1 (9.71)
Usual weekly web-based media use all devices (hours), mean (SD)			28.9 (18.36)
**Sex, n (%)**			
	Male	32 (34)	
	Female	63 (66)	
**Socioeconomic status (n=92), n (%)**			
	Low	15 (16)	
	Medium	26 (27)	
	High	51 (54)	
**Social media platform users, n (%)**			
	Instagram	87 (92)	
	Facebook	69 (73)	
	Snapchat	68 (72)	
	Music streaming apps	68 (72)	
	YouTube	43 (45)	
	Twitter	29 (31)	
	Pinterest	25 (26)	
	Twitch	10 (11)	
**Follow food brands on social media**			
	Frequency (n=91), n (%)	43 (45)	
	Number followed, mean (SD)	0.8 (0.47)	
**Food apps on phone**			
	Frequency (n=91), n (%)	71 (75)	
	Number of apps, mean (SD)	1.9 (1.80)	
**Emails or texts from food brands (n=91), n (%)**			
	None	27 (28)	
	1-5 per week	51 (54)	
	6-10 per week	11 (12)	
	11 or more per week	2 (2)	

### Types of Promotions

Across the sample recordings, there were 4446 food and beverage promotions. Of these 4446 promotions, 2613 (58.77%) were earned media impressions, 732 (16.46%) were on media *owned* by the brand (apps, websites, and pages), and 1101 (24.76%) were paid advertisements. Earned media impressions were mostly seen in content from other nonbrand organization or community sites or pages (eg, meme pages; 2221/2613, 84.99% of earned impressions). A smaller number of earned media impressions were from content shared by a friend (242/2613, 9.26%) or celebrity endorsements (150/2613, 5.74%).

### Promoted Foods and Beverages

The INFORMAS food classification system was used to describe the nature of foods and beverages, as a large number (n=1840) could not be classified using the WHO European nutrient profiling food categories. The highest proportion of promoted foods and beverages was noncore (2579/4446, 58.01%; Table 1). An additional 20.94% (931/4446) of promoted foods and beverages were for fast food restaurants or delivery services that did not promote a specific food or beverage product. The most frequently promoted foods and beverages were fast food restaurants or delivery services (all advertisements combined: 1459/4446, 32.82%), local restaurants or delivery services (all advertisements combined: 618/4446, 13.9%), chocolate and confectionery (539/4446, 12.12%), sugar-sweetened beverages (435/4446, 9.78%), and supermarkets or retailers (all advertisements combined: 371/4446, 8.34%). The most frequently promoted food and beverage brands were McDonald’s (416/4446, 9.36% of all promotions), KFC (184/4446, 4.14%), Coca Cola (137/4446, 3.08%), Uber Eats (135/4446, 3.04%), Starbucks (123/4446, 2.77%), Boost Juice (113/4446, 2.54%), Woolworths (supermarket; 96/4446, 2.16%), Nutella (84/4446, 1.88%), Kit Kat (73/4446, 1.64%), and Coles (supermarket; 73/4446, 1.64%). Together, these top 10 promoted food brands contributed to almost one-third of all promotions.

### Rate of Food and Beverage Promotions

Children were exposed to a median of 17.4 food or beverage promotions each hour on the internet for a total duration of 1.3 minutes per hour (IQR 1-2; Table 3). This included a median of almost 10 earned media impressions per hour. The median rate of promotions for foods that would not be permitted to be marketed based on the WHO nutrient profiling model was 50 times higher than the rate of promotions for foods permitted to be marketed. The food categories with the highest rates of promotions were fast food restaurants or delivery services (company promotions), fast food restaurants or delivery services (promoting unhealthy choices), sugar-sweetened beverages, and chocolates and confectioneries.

**Table 3:** Weighted median rates of web-based food and beverage promotions per hour and by weekly exposures on mobile devices.

Rate of promotions			Weighted median rate per hour (IQR)		Weighted median rate on mobile devices per week (IQR)
Total promotion count			17.4 (10-26)		168.4 (85-289)
**Rate by media type**					
	Earned media	9.9 (6-15)		84.8 (40-177)	
	Paid media	3.7 (1-8)		36.1 (12-75)	
	Owned media	0.6 (0-3)		5.3 (0-36)	
**Rate by World Health Organization Nutrient profiling classification^a,b^**					
	Not permitted	10.0 (5-17)		99.5 (43-159)	
	Company brand only	4.4 (2-8)		37.2 (17-89)	
	Permitted	0.2 (0-1)		3.6 (0-8)	
	Not applicable	0.0 (0-0.4)		0.0 (0-5)	
**Rate by INFORMAS^c^ food classification^d^**					
	Noncore foods	10.1 (5-17)		99.4 (43-159)	
	Miscellaneous	6.4 (2-10)		52.9 (24-99)	
	Core foods	0.0 (0-1)		0.0 (0-8)	
**Rate by food type^d^**					
	Fast food restaurants, no specific product	1.9 (0.6-4)		17.1 (6-46)	
	Fast food restaurants, unhealthy products	1.8 (0.3-4)		16.5 (5-34)	
	Chocolate and confectionery	1.5 (0.3-3)		12.4 (4-29)	
	Sugar-sweetened beverages	0.9 (0-3)		11.6 (0-27)	

^a^Using World Health Organization for Europe Nutrient Profiling Model.

^b^2.59% (115/4446) could not be specified because of unavailable nutrition composition information.

^c^INFORMAS: International Network for Food and Obesity/noncommunicable diseases Research, Monitoring and Action Support.

^d^Using International Network for Food and Obesity/noncommunicable diseases Research, Monitoring and Action Support food classification.

Considering children’s reported usual time on the web on mobile devices, children would be exposed to a median of 168.4 food and beverage promotions on the internet on mobile devices per week for a total duration of 13.2 minutes (IQR 7-27). Children would be exposed to a median of 99.5 food promotions per week on their mobile devices that would not be permitted using WHO nutrient profiling criteria. This includes a median of almost 34 promotions per week for fast food restaurants or delivery services (company only or promoting unhealthy choices), 12.4 promotions for chocolate and confectionery, and 11.6 promotions for sugar-sweetened beverages.

**Figure 1:**
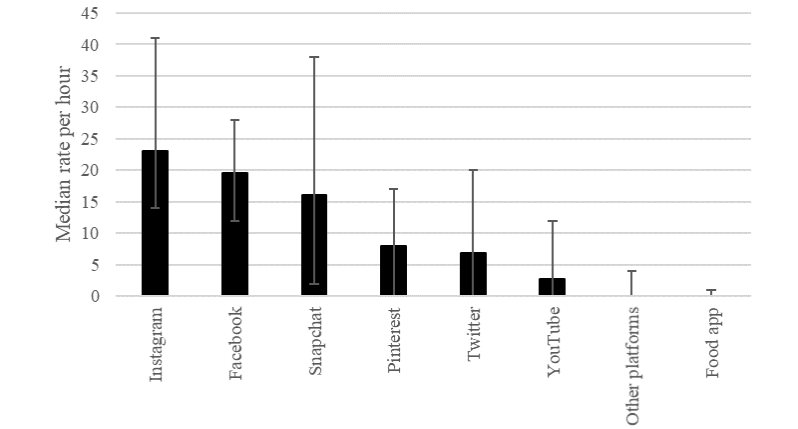
Weighted median rates of web-based food and beverage promotions, by platform. The rates given as a function of time spent on platform, except for food apps, which is given as a function of total time on the web for only those reporting having food apps on phone. Error bars represent IQR. The number of participants visiting each site during the study was 76 for Instagram, 57 for Facebook, 40 for Snapchat, 11 for Pinterest, 22 for Twitter, 58 for YouTube, and 53 for other platforms (apps and websites).

The rates of promotions per hour varied by platform (Kruskal-Wallis H_7_=142.12; *P=*.001; Figure 1). Post hoc pairwise comparisons with Bonferroni correction identified significantly higher rates of promotions on Instagram than on Pinterest, Twitter, YouTube, other platforms (apps and websites), and food apps (all values of *P*=.001). The rates of promotions on Facebook were significantly higher than those on YouTube, other platforms, and food apps (all values of *P=*.001). The rates of promotions on Snapchat were significantly higher than those on other platforms and food apps (all values of *P*=.001).

### Participant Engagement With Promotions

Participant engagement with promotions included *likes*, comments, shares, and clicking on links. Participant engagement with food and beverage promotions was low, with a median of 0.3 (IQR 0-2) overall engagements per hour of time spent on the web or one engagement approximately every 3.4 hours. Each week, participants were estimated to engage with brands a median of 3.9 times, although this was highly variable (IQR 0-21). In the highest decile of engagement, children were estimated to engage with brands at least 47 times per week.

### Predictors of Exposure to Food and Beverage Promotions

Using negative binomial regression, the only factor that was significantly associated with weekly exposure to food and beverage promotions was the amount of time spent on the internet on mobile devices (B=0.54, SE 0.01; *P*<.001; 95% CI 0.03-0.08; Table 4). The incidence rate ratio (IRR) shows that for every 1 hour increase in usual time spent on the internet on mobile devices per week, exposure to food promotions increased by 6% (IRR=1.056, 95% CI 1.028-1.085; *P*<.001). Variables including age, number of accounts followed on social media, number of food or drink brands followed on social media, and number of food apps on mobile devices were not associated with exposure to promotions.

**Table 4:** Negative binomial regression incident rate ratios of the count of weekly exposures to food and beverage promotions on mobile devices.

Independent variable	Incidence rate ratio (95% CI)
Number of food apps	1.09 (0.98-1.21)
Usual weekly time on the web (mobile devices)	1.06 (1.03-1.08)^a^
Any accounts following on social media	1.02 (0.96-1.08)
Age	1.02 (0.85-1.24)
Food brands following on social media	0.95 (0.81-1.11)

^a^*P*=.001.

## Discussion

### Principal Findings

This study exposes Australian children’s exceedingly high exposure to food marketing during their usual time on mobile devices. During each hour that a child spends on the internet on their mobile device, they would see more than 17 food and beverage promotions, equating to 168 promotions per week and 8736 promotions per year. For each hour increase in usual time on the internet on mobile devices per week, children’s exposure to food promotions was found to increase by 6%. Our food marketing exposure estimates are likely to be highly conservative, given that they capture exposures only on mobile devices and not on desktop computers. There is some evidence to suggest that marketing on mobile and nonmobile devices is similar [29]; however, most social media use (where we observed the greatest rates of food promotions) is likely to occur on mobile devices [30]. In contrast, based on some of the most recent data on Australian children’s exposure to food advertising on television, children were estimated to see around 4 food advertisements per hour during broadcast periods that attracted the greatest child audience, including 2 for unhealthy foods [31]. Considering that children aged 0-14 years watch commercial television for an average of 39 minutes per day [32], this would result in 3 exposures to food advertising on television each day or almost 19 exposures per week.

The rates of promotions for unhealthy products were far greater than promotions for healthier choices. Each week, children would be exposed to almost 100 promotions on their mobile devices for foods and beverages that would not be recommended to be marketed to children according to WHO nutrient profiling criteria. In addition, children would see around 17 promotions per week for fast food restaurant companies without a specific product promoted. These could not be appropriately classified using the WHO criteria as recommended to be permitted or otherwise, thus identifying a major limitation of these criteria for classifying food-related brands that should or should not be marketed to children. Although many fast-food outlets sell and promote *healthier* choices, observational data have identified that actual purchase of these healthier choices is infrequent [33]. Nutritional profiling of food companies that sell a range of products of varying nutritional quality, for the purposes of marketing policies, could be applied to the most frequently sold products.

We found that the greatest proportion of food promotion exposures earned media impressions. Although these promotions ostensibly derive from children’s web-based social networks, the brand is often the initiator of earned media messages [34]. Almost all of the earned impressions were from either social media communities or celebrity pages, such as meme pages or web-based influencers. Posts from such third-party pages are increasingly sponsored by brands, allowing brands to access pages’ huge networks of followers on the internet and social cache [35]. Experimental studies have identified that exposure to earned media impressions using web-based influencers leads children to consume more of a promoted snack compared with an alternative nonpromoted product [36]. The inclusion of advertisement disclosure in the web-based promotion had no effect on reducing the intake of the promoted snack. In Australia, the media industry introduced new standards in 2017, requiring marketing to be clearly distinguishable from other content, including promotions by web-based influencers [37]. Another review identified some evidence that exposure to, and engagement with, earned media impressions for alcohol positively increased intentions to consume alcohol and alcohol intake, whereas paid and owned media impressions had no effect [20].

Children in our study engaged with food and beverage promotions by *liking*, commenting or sharing content, or clicking on links in branded content. Although children engaged with relatively few of the overall branded impressions that they were exposed to, this would still equate to a median of almost 4 engagements with web-based food marketing on their mobile devices every week. It is worth noting that viewing impressions of web-based content is an important measure of reach, and even in the absence of engagement, is important to brands. Brands use a mix of promotions designed to increase reach, engagement, and click-throughs [38]. In an earlier cross-sectional survey of young Australian adults, participants’ engagement with, but not their exposure to, web-based marketing for energy drinks was a predictor of their energy drink consumption [39]. The association between engagement with energy drink branded content and consumption in this earlier study was mediated by participants’ subjective norms related to energy drinks, such as the perception that their peers frequently consumed energy drinks. The nature of earned media impressions on social media, which are shared through personal networks, likely boost such perceptions of product acceptability or use by peers [39]. Other qualitative studies have found that young adults are more distrustful of paid advertising on the internet but do not perceive earned media shared by friends as a form of marketing [11,12].

Our finding of the high rates of earned media impressions for unhealthy foods and beverages has major implications for public policy responses to protect children from this marketing. To inform new regulations planned in the United Kingdom to protect children from unhealthy food marketing on television and on the web, the government undertook an impact assessment to evaluate the potential costs and benefits of marketing restrictions [40]. This involved estimating current web-based food marketing exposures, which were based on marketing expenditure and principally considered banner advertisements. The number of advertising impressions (views by children) that were estimated using this approach was approximately five times greater for television advertising than for web-based marketing. Interrogations of the approach used by academics suggested that the impact assessment underestimated children’s actual web-based food marketing exposures by 10-fold [41]. This study supports this finding, whereby Australian children’s web-based food marketing exposures are approximately nine times higher than their exposure to food advertising on television. Our data also clearly refute the premise that banner advertising is a major source of web-based marketing.

To date, most studies seeking to assess the nature and extent of food marketing to children on the internet have been limited to measuring either paid advertising on third-party websites [42] or owned media, including food company websites [43,44] or social media pages [45]. These studies are useful for capturing the range of techniques used by food companies and the techniques that generate the greatest overall user engagement. However, they are unable to quantify children’s exposure to web-based food marketing or the extent of child engagement with brand promotions [9]. There are 2 notable earlier studies that assessed children’s exposure to and engagement with food brand–related content on the web [27,46]. In one study, 101 Canadian children were recorded engaging in 2 social media apps for 5 minutes each on their own mobile device [46]. Similar to this study, based on the extrapolated study data, 12- to 16-year-old children were estimated to be exposed to food promotions an average of 189 times per week. The most promoted products were fast food and sugary drinks. Another study with 12- to 18-year-old from Belgium required participants (n=21) to capture screenshots of food images on their social media platforms over a 1-week period [27]. Aligned with our study, of the branded food images captured, approximately half were earned media impressions, including posts by web-based influencers and celebrities. Inspection of these earned media impressions in this earlier study suggested that these were likely paid marketing.

This study has some limitations. We did not achieve our target sample of 150 children. The final sample was lower than our original anticipated sample because of the substantial time involved in subject recruitment, technical errors with video uploads, and difficulties in obtaining complete data from participants. In an earlier pilot study, we had achieved a response rate of approximately 50%. This was substantially reduced in the main study, as we introduced a minimum threshold for daily video upload time. Surprisingly, we did not find significant associations between the number of overall accounts or food accounts that children followed on the internet or the number of food apps they had on their device and their marketing exposures. The CIs around the IRRs for these variables were wide, and future studies may be more adequately powered to detect significant associations. The minimum threshold for daily video upload of 30% of the usual time spent on the web on mobile devices meant that we did not capture all time spent on the internet on mobile devices. However, a comparison of the rates of food marketing exposures across data sets manipulated to include between 30% and 80% of the usual time spent on the internet recorded found there to be excellent reliability across the data sets (data not shown). Finally, the recruitment of children through the market research company survey panel may affect the generalizability of the findings to a broader population. However, this approach allowed us to capture a national sample, with representation from metropolitan and regional areas. This panel recruits multiple web-based and offline sources to recruit a broad spectrum of participants.

Opportunities for protecting children from web-based food marketing span legislative or regulatory controls, industry codes of practice for responsible marketing, and interventions that operate on an individual level to block exposure to marketing content. Internationally, some governments have introduced or are introducing restrictions on unhealthy food marketing to children on the web. As mentioned previously, the UK government announced plans to introduce a ban on all web-based marketing of unhealthy foods and beverages by 2022, as part of its national obesity prevention strategy [47]. In other jurisdictions, including Brazil, Peru, and Quebec, Canada, food marketing regulations apply comprehensively across all or most settings and media platforms, including digital media [48]. These latter regulations preclude advertising *directed to children*. The potential for marketers to circumvent this provision of restricting only marketing that is of specific appeal to children may limit the impact of such regulations.

To date, food industry codes of practice for responsible marketing largely fail to cover the types of web-based platforms that children use or the types of marketing they see or engage with on these platforms. For example, the International Food & Beverage Alliance Global Policy on Marketing Communications to Children only applies to media primarily directed to children aged <12 years and only applies to company-owned websites [49]. It does not, therefore, apply to any form of marketing on social media platforms, including company-owned pages. More recently, the digital media industry introduced responsible food marketing codes of practice. In October 2020, Google introduced restrictions on unhealthy food and beverage advertising for children aged <18 years in the United Kingdom and European Union [50]. This code requires advertisers to self-declare if they are using a web-based account to promote unhealthy foods or beverages, after which Google will block advertising from this account to children through its network of websites, videos, and apps. Although ostensibly this represents exceptional leadership by the media industry on this issue, previous restrictions on data mining and behavioral targeting of advertising to children aged <13 years on social media sites in the United Kingdom have been ineffective [51]. Given the global nature of Google, there is also no discernible reason why the policy should not be extended to all jurisdictions, rather than only where there is a threat or promise of government intervention. Our study identified significantly higher rates of exposure to food marketing on Instagram, Facebook, and Snapchat, signaling opportunities for these platforms to self-regulate to protect children from unhealthy food and beverage promotions. These platforms already self-regulate advertising content for tobacco products, although this is limited to restricting paid advertising. Research evidence shows that web-based promotion of tobacco products continues unabated through web-based influencers [52].

Finally, ad blockers and antitracking apps are available to block paid advertising and web-based tracking, which enables targeted advertising, on desktop computers and mobile devices. This includes software to block advertising and sponsored posts on social media. Some paid versions of social media platforms, such as YouTube Premium, also offer ad-free content. Although this study and others have highlighted that paid advertising is only a minority of the marketing impressions that children see on the web, this software may still be useful in reducing up to one-fifth of the web-based food marketing that children are exposed to. However, it is likely that widespread uptake of ad blockers would lead brands to invest further in earned and owned media, thereby further increasing those types of media impressions.

### Conclusions

Using real-time monitoring over a 3-day period, this study identified that Australian children are exposed to an outstanding volume of web-based food marketing on their mobile devices. This marketing is predominantly for unhealthy products and is shared through web-based communities. Children typically engage in web-based marketing multiple times each week. This exposure to, and interaction with, brand content that is seemingly endorsed by peers or web-based communities likely heightens the effects of marketing on children’s brand attitudes and consumption behaviors. Governments and the media industry can and have designed policies to protect children from this marketing. The rapid acceleration and the use of data analytics and technologies used to capture personal data for targeted marketing is outstripping current legislation and policies for appropriate marketing regulations and related ethical concerns. To ensure that such policies are effective, they need to extend beyond paid advertising to paid content in posts generated through web-based communities, influencers, and celebrities.

## Abbreviations

INFORMAS: International Network for Food and Obesity/noncommunicable diseases Research, Monitoring and Action Support
IRR: incidence rate ratio
WHO: World Health Organization
